# Pulmonary Abscess With Contiguous Extension to the Chest Wall in an Immunocompetent Young Woman: A Case Report

**DOI:** 10.7759/cureus.113629

**Published:** 2026-07-29

**Authors:** Iara Ferreira, Daniela Soares, Beatriz Vitó Madureira, Andreia M Teixeira

**Affiliations:** 1 Internal Medicine, Unidade Local de Saúde de Entre Douro e Vouga, Santa Maria da Feira, PRT

**Keywords:** chest wall extension, immunocompetent patient, lung abscess, pulmonary cavitation, thoracic infection

## Abstract

Lung abscesses are localized necrotizing infections of the pulmonary parenchyma that rarely extend into the chest wall. We report the case of a previously healthy 21-year-old veterinary medicine student who presented with fever and painful left infraclavicular swelling that became more prominent with coughing, despite the absence of significant respiratory symptoms. Laboratory evaluation revealed marked neutrophilic leukocytosis and an elevated C-reactive protein level. Contrast-enhanced chest computed tomography (CT) demonstrated left upper lobe consolidation with a cavitary lesion containing an air-fluid level and extending through the first intercostal space into the anterior chest wall and subpectoral tissues. An extensive microbiological investigation excluded relevant alternative infectious etiologies, although respiratory cultures yielded findings of uncertain clinical significance. Percutaneous drainage was considered but deferred because of the limited drainable fluid component. The patient completed four weeks of intravenous antimicrobial therapy, with progressive clinical, laboratory, and radiological improvement. At the three-month follow-up, chest CT confirmed complete resolution of both the pulmonary and chest wall lesions. This case highlights chest wall swelling as an uncommon presentation of lung abscess and emphasizes the importance of cross-sectional imaging, cautious interpretation of respiratory cultures, and individualized multidisciplinary management.

## Introduction

A lung abscess is a localized necrotizing infection of the pulmonary parenchyma characterized by the formation of a pus-containing cavity. It most commonly results from aspiration of oropharyngeal secretions and may also occur in association with necrotizing pneumonia. Recognized predisposing factors include impaired consciousness, poor dentition, dysphagia, structural lung disease, bronchial obstruction, and immunosuppression. Patients typically present with fever, productive cough, chest pain, and constitutional symptoms, although clinical manifestations may be nonspecific [1].

Potential complications include pleural involvement, empyema, bronchopleural fistula, hemoptysis, and sepsis [1]. Direct extension of a lung abscess into the chest wall and adjacent soft tissues has been described in isolated case reports [2,3]. We present the case of an otherwise healthy 21-year-old woman with fever and painful infraclavicular swelling but without significant respiratory symptoms. Imaging revealed a left upper lobe lung abscess extending through the first intercostal space into the anterior chest wall and subpectoral tissues. This case highlights an uncommon presentation of lung abscess and emphasizes the importance of cross-sectional imaging and multidisciplinary management.

## Case presentation

A 21-year-old woman with no relevant medical history presented to the emergency department with a two-week history of painful left infraclavicular swelling and a four-day history of fever. She was a veterinary medicine student with frequent exposure to horses. She denied smoking, illicit drug use, recent travel, aspiration, dyspnea, or significant cough.

On admission, she was hemodynamically stable, with a temperature of 38.0°C and an oxygen saturation of 98% on room air. Physical examination revealed a soft left infraclavicular swelling that became more prominent with coughing and decreased breath sounds over the left lung apex. Laboratory testing showed neutrophilic leukocytosis of 31.5 × 10⁹/L and an elevated C-reactive protein level of 346.1 mg/L (Table 1).

**Table 1:** Laboratory parameters at admission Initial laboratory evaluation demonstrated marked leukocytosis, thrombocytosis, mild anemia, and elevated inflammatory markers, with preserved renal and hepatic function. Reference intervals may vary slightly depending on laboratory standards.

Parameter		Result	Unit	Reference Range
Hemoglobin		11.4	g/dL	12.0 - 16.0
White blood cells		31.5	x10⁹/L	4.0 – 11.0
Platelets		499	x10⁹/L	150 - 450
International normalized ratio (INR)		1.1	-	-
Urea		24	mg/dL	15 - 40
Creatinine		0.7	mg/dL	0.6 – 1.1
Sodium		137	mmol/L	136.0 – 145.0
Potassium		4.7	mmol/L	3.5 – 5.1
Total bilirubin		0.42	mg/dL	0.20 – 1.20
Aspartate aminotransferase (AST)/alanine aminotransferase (ALT)		14/13	U/L	5 - 34 / 0 - 55
Lactate dehydrogenase (LDH)		167	U/L	125 - 220
Erythrocyte sedimentation rate		36	mm	0.0 – 12.0
Procalcitonin		7.42	ng/mL	< 0.1
C-reactive protein (CRP)		346.1	mg/L	0.0 – 5.0

**Figure 1:**
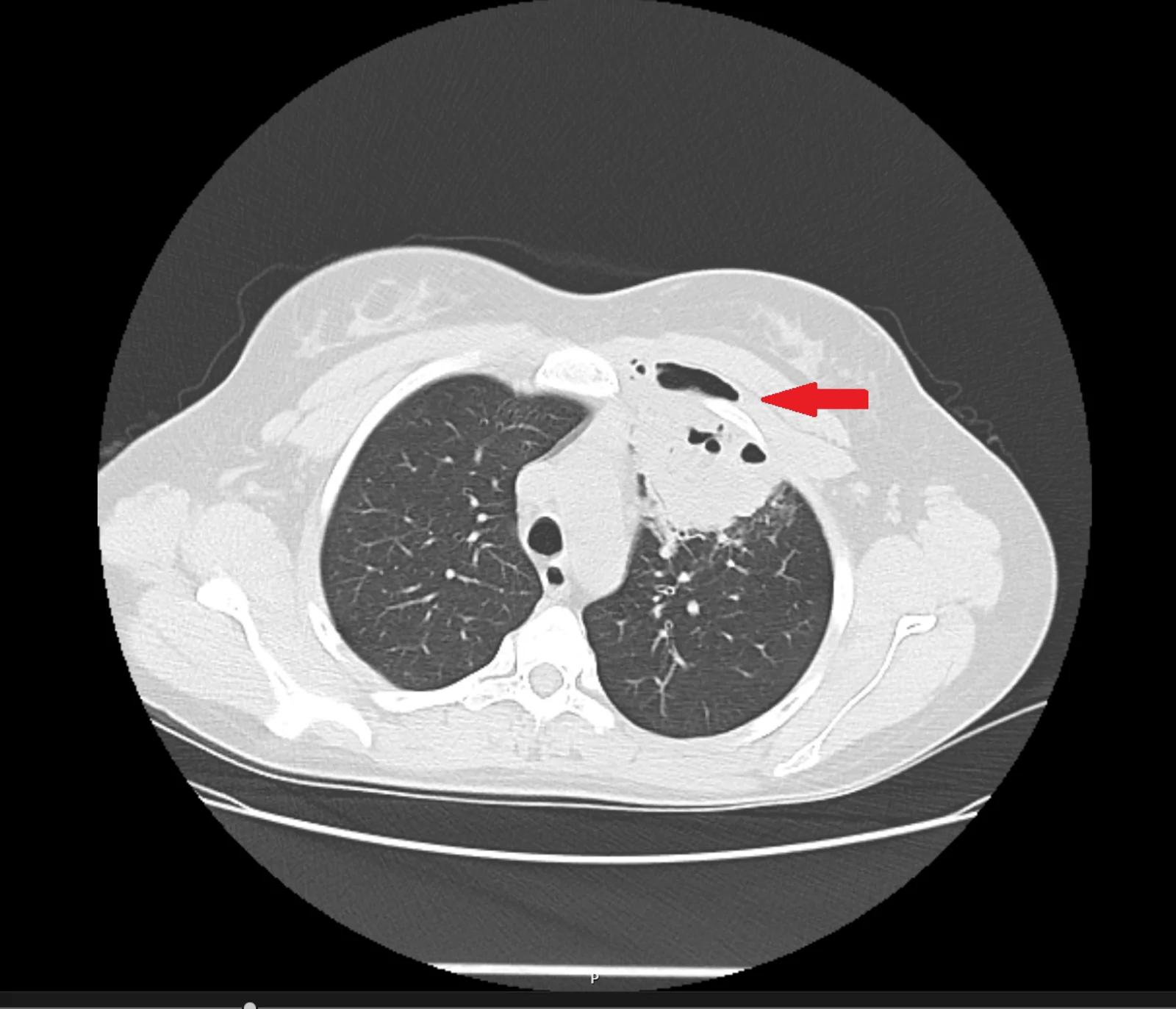
Contrast-Enhanced Chest Computed Tomography Showing a Left Upper Lobe Lung Abscess With Chest Wall Extension Axial contrast-enhanced chest computed tomography image demonstrating consolidation in the anterior segment of the left upper lobe with a cavitary lesion containing an air-fluid level (red arrow). The lesion extends through the first intercostal space into the anterior chest wall and subpectoral soft tissues, consistent with a lung abscess with contiguous fistulous extension.

Contrast-enhanced chest computed tomography (CT) demonstrated consolidation in the anterior segment of the left upper lobe associated with a 4.4 × 3.5 cm cavitary lesion containing an air-fluid level (Figure 1).

The lesion extended through the first intercostal space into the anterior chest wall and subpectoral soft tissues, consistent with a lung abscess with fistulous extension into the chest wall. After blood cultures and other microbiological samples were obtained, empiric treatment with amoxicillin-clavulanate was initiated. Because of persistent fever, antibiotic therapy was escalated to piperacillin-tazobactam. Percutaneous drainage was considered but was not performed after reassessment by interventional radiology.

An extensive diagnostic workup was performed to identify the causative pathogen and exclude alternative infectious etiologies (Table 2).

**Table 2:** Immunologic and microbiological workup Summary of immunologic and microbiologics investigations performed during hospitalization. Immunologic testing was unremarkable except for a mild elevation in immunoglobulin A. Blood cultures, HIV testing, mycobacterial studies, and the zoonotic panel were negative. Bronchoalveolar lavage yielded scant penicillin-susceptible *Staphylococcus aureus*, while bronchial washing grew *Candida albicans*.

Parameter		Result	Unit	Reference Range
Immunoglobulin A		424	mg/L	65 - 421
Immunoglobulin G		1201	mg/L	552 - 1631
Immunoglobulin M		95	mg/L	33 - 293
Immunoglobulin E		10.5	KU/L	0 - 120.0
Human immunodeficiency virus (HIV) serology		Negative	_	_
Blood cultures (two aerobic sets + two anaerobic sets)		Negative	_	_
Sputum acid-fast bacilli smears, three samples		Negative	_	_
Sputum mycobacterial cultures		Negative	_	_
Bronchoalveolar lavage acid-fast bacilli smear by Ziehl-Neelsen staining		Negative	_	_
Bronchoalveolar lavage Mycobacterium tuberculosis complex DNA testing		Negative	_	_
Bronchoalveolar lavage mycobacterium culture		Negative	_	_
Bronchoalveolar lavage bacterial culture		Scant penicillin-susceptible Staphylococcus aureus and isolated Candida albicans	_	_
Zoonotic infection panel		Negative	_	_
Respiratory virus polymerase chain reaction		Negative	_	_
Urinary Legionella pneumophila antigen		Negative	_	_
Urinary Streptococcus pneumoniae antigen		Negative	_	_

Blood cultures and testing for acid-fast bacilli, human immunodeficiency virus, zoonotic infections, Actinomyces, Nocardia, and the Mycobacterium tuberculosis complex were negative. Bronchoscopy revealed no endobronchial abnormalities. Bronchoalveolar lavage culture yielded scant penicillin-susceptible *Staphylococcus aureus*, while bronchial washing grew *Candida albicans*. These findings were interpreted cautiously because their etiological significance was uncertain. Nevertheless, fluconazole was added after multidisciplinary review.

**Figure 2:**
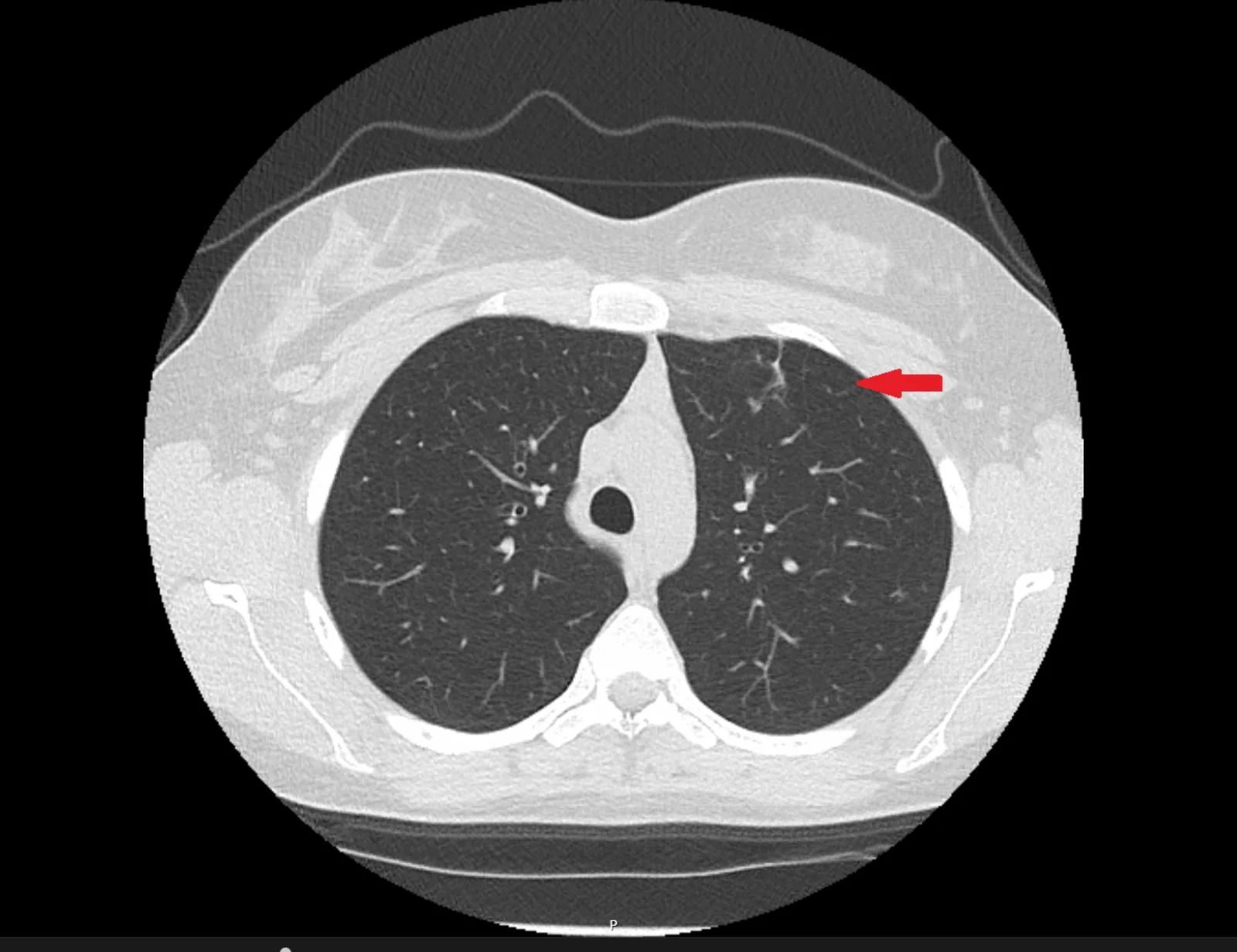
Three-Month Follow-Up Chest Computed Tomography Axial chest computed tomography obtained three months after treatment showing resolution of the left upper lobe cavitary lesion and chest wall extension, with minimal residual linear subpleural scarring at the previous site of infection (red arrow).

The patient completed four weeks of intravenous antimicrobial therapy, with progressive clinical, laboratory, and radiological improvement. She remained hemodynamically stable and did not require supplemental oxygen throughout hospitalization. At the three-month outpatient follow-up, she was asymptomatic, and follow-up chest CT imaging confirmed complete resolution of the pulmonary and chest wall lesions (Figure 2).

## Discussion

This case describes a rare complication of pulmonary infection in a young, immunocompetent woman without recognized risk factors for lung abscess. She had no history of aspiration, impaired consciousness, underlying pulmonary disease, smoking, illicit drug use, or immunosuppression. Despite marked systemic inflammation and extensive anatomical involvement, respiratory symptoms were minimal. Fever and painful infraclavicular swelling were the predominant manifestations, and the swelling became more prominent with coughing. This atypical presentation could initially suggest a musculoskeletal, soft tissue, or neoplastic process rather than a pulmonary infection.

Contrast-enhanced chest CT was essential for establishing the diagnosis and defining the anatomical extent of the disease. It demonstrated a cavitary lesion with an air-fluid level within left upper lobe consolidation, consistent with a lung abscess, and revealed contiguous extension through the first intercostal space into the anterior chest wall and subpectoral tissues. These findings explained the cough-related prominence of the swelling and the reduced breath sounds over the left lung apex.

Chest wall extension of intrathoracic infection is classically described in empyema necessitans, whereas imaging in this case favored direct extension from a pulmonary parenchymal cavity rather than from a dominant pleural collection [2]. This distinction is clinically relevant because the origin and anatomical pathway of infection may influence source-control decisions. Direct extension of a lung abscess into the chest wall has been described in isolated case reports, some of which required percutaneous drainage, surgical debridement, or pulmonary resection [2,3]. In contrast, the present patient achieved complete resolution with medical treatment alone.

Lung abscess and necrotizing pneumonia are related manifestations of suppurative pulmonary parenchymal infection and may be associated with necrosis, cavitation, persistent inflammation, and pleural complications [1,4]. The differential diagnosis of a cavitary pulmonary lesion includes mycobacterial and fungal disease, inflammatory disorders, localized pleural infection, and cavitating malignancy [1].

The patient’s frequent exposure to horses was epidemiologically relevant because *Rhodococcus equi *can cause cavitary pulmonary infection, predominantly in immunocompromised individuals but also in immunocompetent hosts [5]. Her veterinary background therefore justified an expanded microbiological investigation. Testing for zoonotic infections, *Mycobacterium*, *Actinomyces*, and *Nocardia* was negative. Bronchoscopy also revealed no endobronchial abnormality suggestive of a foreign body, tumor, or another cause of bronchial obstruction. These findings reduced the likelihood of the main alternative diagnoses, although the absence of direct sampling from the abscess prevented definitive microbiological identification.

The respiratory culture results required cautious interpretation. Bronchoalveolar lavage yielded scant penicillin-susceptible *Staphylococcus aureus*. This organism is a recognized cause of lung abscess and necrotizing pneumonia and may be associated with severe necrotizing and cavitary pulmonary infection [1,4]. It was therefore a plausible causative pathogen. However, its etiological role could not be confirmed because growth was scant, blood cultures were negative, respiratory sampling was performed after antimicrobial therapy had been initiated, and no specimen was obtained directly from the pulmonary cavity or chest wall component. Accordingly, *S. aureus* should be regarded as a possible rather than definitively established causative organism.

The isolation of *Candida albicans* from bronchial washing was also of uncertain clinical significance. Recovery of Candida species from respiratory secretions usually represents airway colonization and rarely warrants antifungal therapy [6]. A definitive diagnosis of *Candida* pneumonia generally requires histopathological evidence of tissue invasion, and treatment decisions should not be based solely on a positive respiratory culture [6]. Invasive pulmonary candidiasis would also be unusual in an immunocompetent patient without candidemia or evidence of disseminated fungal infection [6]. Fluconazole was added after multidisciplinary review; however, its concomitant administration with broad-spectrum antibacterial therapy precludes any conclusion regarding the contribution of C. albicans to the infection or the independent benefit of antifungal treatment.

Systemic antimicrobial therapy is the mainstay of lung abscess treatment. Empirical treatment should provide broad coverage of the mixed aerobic and anaerobic organisms commonly implicated in these infections, with subsequent adjustment according to microbiological findings and clinical response. Treatment duration should be individualized according to clinical, laboratory, and radiological evolution [1]. In this case, amoxicillin-clavulanate was initiated after microbiological samples were obtained. Persistent fever prompted escalation to piperacillin-tazobactam, followed by progressive clinical, laboratory, and radiological improvement during four weeks of intravenous antimicrobial therapy.

Percutaneous or endoscopic drainage and surgical treatment may be considered when systemic antimicrobial therapy fails to achieve clinical or radiological improvement [7]. Because of the chest wall extension, percutaneous drainage was considered early. However, reassessment by interventional radiology demonstrated only a small drainable fluid component, and the procedure was not performed. The patient remained hemodynamically stable, maintained normal oxygenation, and showed sustained improvement with medical therapy alone. Complete resolution of both the pulmonary and chest wall lesions at three months supports conservative treatment as a reasonable option in carefully selected, clinically stable patients who demonstrate a favorable response and can undergo close clinical and radiological follow-up.

The principal limitation of this case was the absence of direct microbiological sampling from the pulmonary cavity or chest wall component. Consequently, the causative pathogen could not be established definitively. In addition, the concomitant use of antibacterial and antifungal agents prevents assessment of their individual contributions to the clinical response. Nevertheless, the marked inflammatory response, characteristic imaging findings, exclusion of relevant alternative diagnoses, response to antimicrobial therapy, and complete radiological resolution support the diagnosis of a lung abscess of probable bacterial origin with direct extension into the chest wall.

This case emphasizes that painful chest wall swelling associated with fever should not automatically be regarded as a superficial process, particularly when its prominence changes with coughing. Early contrast-enhanced CT is important for identifying the origin and anatomical extent of the infection and for guiding subsequent management [1]. Respiratory culture results should be interpreted in conjunction with organism burden, sampling conditions, imaging findings, and clinical evolution rather than regarded as definitive evidence of causation in isolation [6].

## Conclusions

Direct extension of a lung abscess through an intercostal space into the chest wall is a rare complication, particularly in a young, immunocompetent patient without conventional predisposing factors. Fever associated with painful infraclavicular swelling may therefore indicate an underlying pulmonary infection, even in the absence of respiratory symptoms.

Contrast-enhanced CT is essential for identifying the pulmonary origin and anatomical extent of the infection and for guiding source-control decisions. Microbiological findings from respiratory samples should be interpreted cautiously and in conjunction with the clinical and radiological findings. Although invasive drainage should be considered in patients with an inadequate response or complications requiring source control, prolonged antimicrobial therapy with close clinical and radiological follow-up may achieve complete resolution in selected clinically stable patients.
